# Post-Translational Modifications Control Phase Transitions of Tau

**DOI:** 10.1021/acscentsci.4c01319

**Published:** 2024-11-13

**Authors:** Wyatt C. Powell, McKinley Nahum, Karl Pankratz, Morgane Herlory, James Greenwood, Darya Poliyenko, Patrick Holland, Ruiheng Jing, Luke Biggerstaff, Michael H. B. Stowell, Maciej A. Walczak

**Affiliations:** † Department of Chemistry, University of Colorado, Boulder, Boulder, Colorado 80309, United States; ‡ Department of Molecular, Cellular and Developmental Biology, University of Colorado, Boulder, Boulder, Colorado 80309, United States

## Abstract

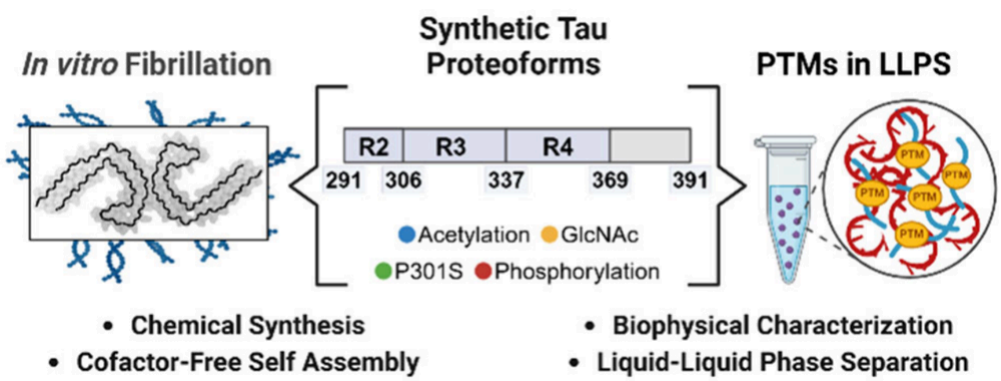

The self-assembly of Tau into filaments, which mirror the structures observed in Alzheimer’s disease (AD) brains, raises questions about the role of AD-specific post-translational modifications (PTMs) in the formation of paired helical filaments (PHFs). To investigate this, we developed a synthetic approach to produce Tau(291–391) featuring *N*-acetyllysine, phosphoserine, phosphotyrosine, and *N*-glycosylation at positions commonly modified in post-mortem AD brains. Using various electron and optical microscopy techniques, we discovered that these modifications generally hinder the *in vitro* assembly of Tau into PHFs. Interestingly, while acetylation’s effect on Tau assembly displayed variability, either promoting or inhibiting phase transitions in cofactor-free aggregation, heparin-induced aggregation, and RNA-mediated liquid–liquid phase separation (LLPS), phosphorylation uniformly mitigated these processes. Our observations suggest that PTMs, particularly those situated outside the rigid core, are pivotal in the nucleation of PHFs. Moreover, with heparin-induced aggregation leading to the formation of heterogeneous aggregates, most AD-specific PTMs appeared to decelerate aggregation. The impact of acetylation on RNA-induced LLPS was notably site-dependent, whereas phosphorylation consistently reduced LLPS across all proteoforms examined. These insights underscore the complex interplay between site-specific PTMs and environmental factors in modulating Tau aggregation kinetics, highlighting the role of PTMs located outside the ordered filament core in driving the self-assembly.

## Abstract 2

Synthetic peptide fragments of Tau self-assemble into fibrils mirroring paired helical filaments. Post-translational modifications in the ordered core control aggregation and phase transitions.

## Introduction

**Figure 1:**
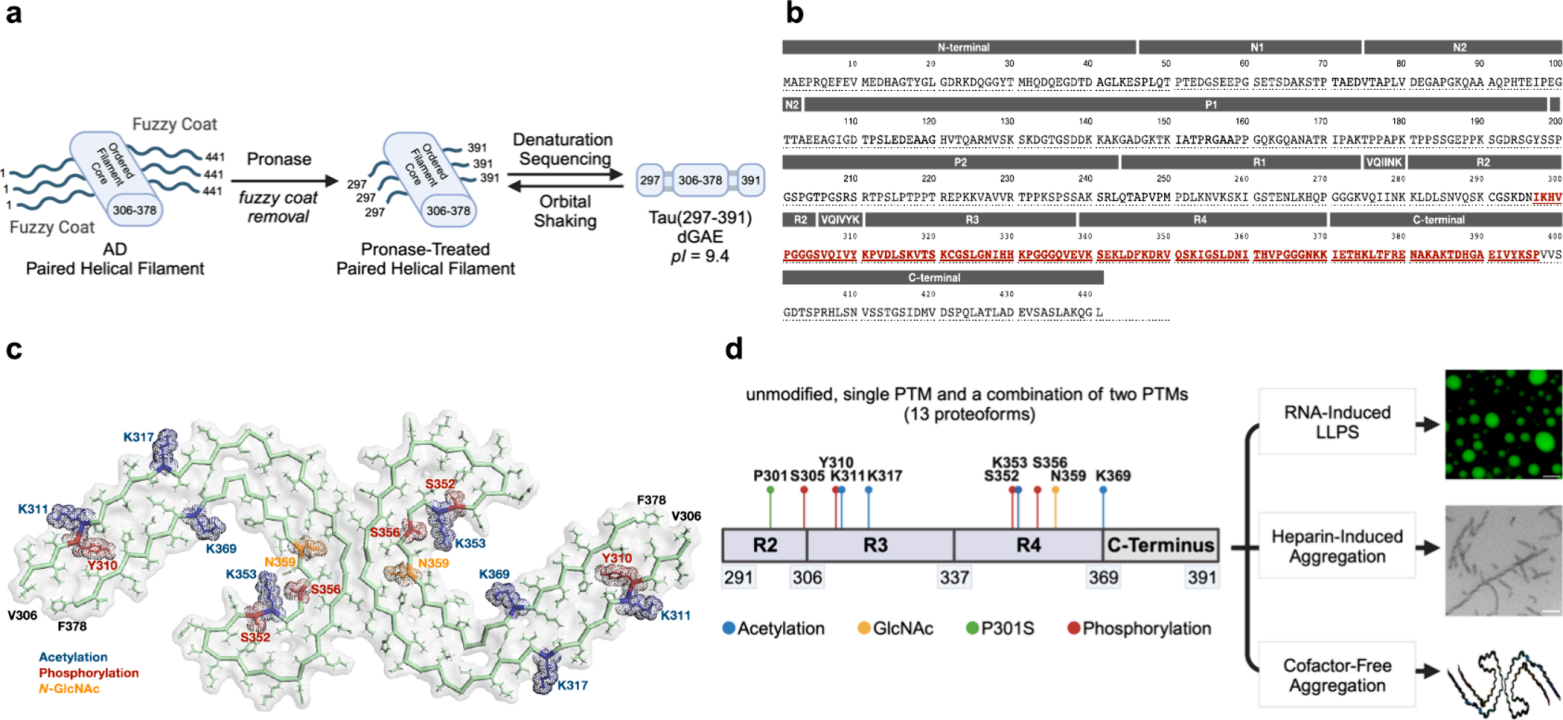
(a) Identification of the PHF core sequence in AD patients. Alzheimer’s disease paired helical filaments consist of a rigid filament core and a fuzzy coat that contains the N- and C-terminal regions. Cleavage of the fuzzy coat from AD paired helical filaments with Pronase releases the proteolytically stable filament core.^1,2^ (b) Primary sequence of 2N4R Tau with the 297–391 peptide region highlighted in red. (c) Mapping of selected PTMs onto the AD core structure (PDB: 5O3L). (d) An overview of the study plan.

Deposits of microtubule-associated protein Tau are implicated in the onset and progression of several neurodegenerative diseases known as tauopathies.^3^ Primary tauopathies such as corticobasal degeneration (CBD) are characterized by neuronal and glial inclusions. In secondary tauopathies including Alzheimer’s disease (AD), Tau in neurofibrillary tangles (NFTs) is found together with amyloid-β plaques. Tau filaments from different patients with the same disease including AD, chronic traumatic encephalopathy (CTE), CBD, and Pick’s disease reproducibly adopt the same unique molecular structures.^4−7^ These disease-specific polymorphs may represent low energy conformations of Tau and, together with environmental factors, likely play a role in the structural diversity and filament propagation.^8−11^ However, many *in vitro* and mouse model filaments create multiple Tau polymorphs and do not resemble the structures observed in human tauopathies.^12−14^ Only recently aided by high-throughput cryo-electron microscopy (cryo-EM), short fragments of Tau have been shown to self-assemble into the AD and CTE filaments, but these peptides also form many other structures under similar conditions (Figure 1).^15−19^

The factors that assist or control folding of full-length Tau into single disease-specific polymorphs remain unclear. In understanding the structural polymorphism of Tau, post-translational modifications (PTMs) that alter the charge and hydrophobicity of amino acid side chains need to be considered.^20^ Tau filaments from AD and other tauopathies are heavily modified with PTMs, and site-specific modifications unique to the disease-associated filaments are also observed.^21,22^ It has been suggested that depending on the location of the modification relative to the fibril core, PTMs can alter the primary structure of Tau and drive folding into specific structures.^23−26^ Alternatively, Tau filaments may be nucleated by environmental factors, and site-specific PTMs occur due to the pre-established conformation of the filament. Hydrophobic and nonproteinaceous cofactor densities are observed in several types of Tau filaments, indicating strong interactions between various cellular components and Tau aggregates.^27−29^ Seeding of Tau in cells with AD- and CBD-derived brain seeds does not recapitulate the structures of the added brain seeds, and a similar effect is observed for α-synuclein filaments from multiple systems atrophy brain seeds.^30−32^ Understanding the role of these factors may yield a better insight into the mechanism of the disease and potentially provide a platform to fine-tune diagnostic tools (e.g., antibodies) or even novel therapeutic strategies that capitalize on aberrant PTMs. Here, we describe synthetic, structural, and biophysical studies that comprehensively evaluate the role of each PTM in the ordered AD region and demonstrate the first example of a fully synthetic AD fibril produced by chemical means. This study also establishes a relationship between the position of the PTM and its effect on phase transitions. We demonstrate the synergy between two complementary PTMs that can be challenging to decipher with other methods, and we show that some PTMs dominate the biophysical behavior of Tau.

## Results

To probe the role of post-translational modifications in Tau phase transitions, we used chemical protein synthesis (Figure 1). We aimed to understand how site-specific modifications (acetylation, phosphorylation, and glycosylation) modulate AD filament structure, regulate aggregation, and regulate liquid–liquid phase separation (LLPS). We hypothesized that AD-specific PTMs can facilitate the nucleation of Tau paired helical filaments (PHFs) *in vitro*. The Tau region of interest was selected based on the published study that identified Tau(297–391) as the sequence that recapitulates the AD fold *in vitro*(15−19) and our prior synthetic work, which indicated a strategically advantageous synthetic disconnection at C291 to produce a peptide fragment Tau(291–391).^45^ We selected three PTMs that are linked to Tau physiology and dysfunction:

(A) Acetylation of Tau filaments occurs in the region that forms the rigid fibril core and usually overlaps with ubiquitination sites.^24,33^ For this study, we selected four individual acetylated positions (AcK311, AcK317, AcK353, and AcK369), which have been observed with high patient frequency in AD, globular glial tauopathy (GGT), frontotemporal dementia with parkinsonism-17 (FTDP-17), CBD, progressive supranuclear palsy (PSP), and Pick’s disease.^24,33^
(B) Phosphorylation of Tau filaments is typically observed outside of the ordered fibril core, and the phosphorylation sites within the selected region are likely important for regulating microtubule binding. In our study, we selected four phosphorylation sites (pS305, pY310, pS352, and pS356) that have all been observed with various frequencies in AD, with pS305 being the most common AD-specific phosphorylation in this region.^33^ Additionally, two positions (pS356 and pS352) are also important for regulating microtubules binding. Three of these sites are modified serine sites, and one unique position is phosphorylated tyrosine.
(C) *N*-Glycosylation of Tau only occurs in AD patients but not in healthy controls, and it might be responsible for *in vivo* neurofibrillary tangle formation.^34−36^ We chose to investigate *N*-acetyl glucosamine at GlcNAcN359 as one of the two potential glycosylation sites.
(D) To better understand the interplay between two orthogonal PTMs (phosphorylation or acetylation) and *N*-glycosylation,^37^ we included two additional proteoforms designated as GlcNAcK359 + AcK353 and GlcNAcN359 + pS305.
(E) Finally, to complement these studies, we selected another relevant modification (P301S) that is linked to inherited FTDP-17 and CBD.^38^ In this mutation, we used only unmodified protein chain, which served as the reference point and a benchmark, together with the unmodified (WT) Tau(291–391) peptide. Together, the described study includes 13 unique proteoforms.

### A. Synthesis of Tau(291–391)

**Figure 2:**
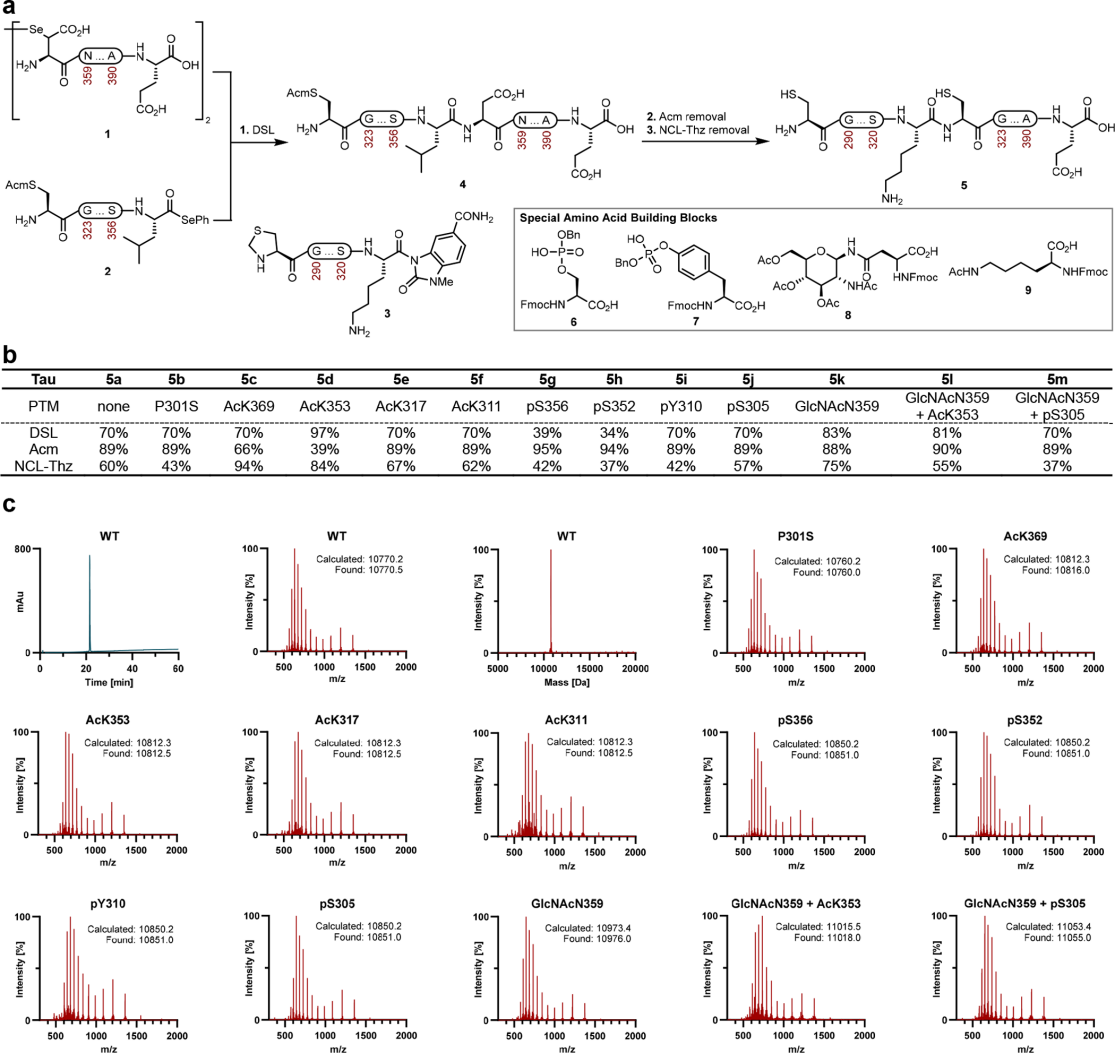
Chemical synthesis of Tau(291–391). (a) General synthetic scheme outlining the key synthetic peptide fragments used to assemble Tau(291–391). Reaction conditions: 1. DSL: 1a. 6 M Gnd·HCl, 100 mM Na_2_HPO_4_, pH 6.2, 23 °C, 1 h; 1b. extraction with hexane; 1c. adjust to 2% (v/v) hydrazine hydrate, pH 7.4, 23 °C, 10 min; 1d. adjust to 83 mM TCEP·HCl, 8 mM DTT, pH 5.3, 23 °C, 30 min. 2. Acm removal: AgOAc (30 equiv), AcOH:H_2_O (1:1), 23 °C, 6 h; or PdCl_2_ (5 equiv), MgCl_2_ (15 equiv), 6 M Gnd·HCl, 0.1 M Na_2_HPO_4_, pH 7.2, 37 °C, 2 h. 3. NCL-Thz removal: 200 mM MPAA, 6 M Gnd·HCl, 200 mM Na_2_HPO_4_, 20 mM TCEP·HCl, pH 7.0, 23 °C, 4 h; adjust to 200 mM MeONH_2_·HCl, pH 4.0, 23 °C, 4 h. (b) Summary of reactions yields for each Tau(291–391) proteoform with individual PTMs. DSL refers to step 1 in Figure 2a and indicates the isolated yield of peptide fragment **4** after deselenization. Acm refers to the yields of an isolated intermediate after Acm removal. NCL-Thz removal refers to the yields of purified **5** obtained after NCL with fragments **3**, followed by deprotection of the final product with MeONH_2_. (c) LC-MS data for unmodified Tau(291–391) (first three entries) and ESI-MS data for the remaining Tau(291–391) proteoforms. LC gradient for WT: 5–65% MeCN/H_2_O/0.05% TFA over 60 min, 4.6 × 100 mm, 4 μm, 100 Å, InfinityLab Poroshell 120 EC-C18 column (214 nm UV detection).

To access the selected modifications of Tau, we used chemical protein synthesis (Figure 2). In our approach, we divided the 101-mer peptide into three segments and merged them using the native chemical ligation (NCL) reaction.^39^ Because the Tau(291–391) region contains an internal cysteine residue, we selected the K322–C321 junction for the last ligation step. This synthetic step would also be conducted using the tandem NCL-Thz removal protocol.^40^ For the first ligation site, we selected the L357–D358 junction using the diselenide-selenoester ligation (DSL)-deselenization protocol.^41−43^ Both the K322–C321 and L357–D358 ligation sites have been utilized in previous syntheses.^44−47^ Using these disconnections, three peptide segments were envisioned: Tau(291–321), Tau(322–357), and Tau(358–391). These segments were designed to contain the relevant PTMs and could be assembled in a combinatorial fashion.

Several solid-phase peptide synthesis (SPPS) methods are crucial to preparing the peptide segments with post-translational modifications. Segment **1** corresponding to Tau(358–391) was prepared on Wang-peg resin to generate the C-terminal carboxylic acid, and the diselenide intermediate was formed from a PMB-protected selenoether under oxidative conditions (TFA, DMSO).^43^ The unmodified and AcK369 diselenides were relatively stable to the typical synthetic manipulations, but the GlcNAcN359 analogue was prone to deselenization and was lyophilized immediately after HPLC purification and protected from light to minimize material loss. Segment **2**, which corresponds to Tau(322–357), contains an N-terminal Acm cysteine and a C-terminal selenoester. Segment **2** was prepared on HMPB ChemMatrix resin; after SPPS, the protected peptide acid was selectively detached from the resin, and a phenyl selenoester was installed at the C-terminus using DPDS/P(*n*-Bu)_3_.^48^ Segment **3**, which corresponds to Tau(291–321), contained an N-terminal Thz protective group and has a C-terminal Nbz linker, which behaves like a thioester during the NCL step.^49^ We note that the phosphorylated amino acid building blocks were quantitatively coupled onto the growing chain using 2.5 equiv of the reagent and a combination of HATU/Oxyma/DIPEA for 16 h.^50^ The selenoaspartic acid building block used in the preparation of fragment **1** was coupled overnight using 2 equiv of the reagent, but with DIC/Oxyma for 16 h. The SPPS methods were effective for the preparation of all the peptide segments with PTMs in similar yields and purity (for details, see the Supporting Information).

The forward synthesis of the Tau(291–391) peptides was completed in a combinatorial manner. Segments **1** and **2** were ligated by the one-pot method using the diselenide-selenoester-ligation-deselenization, and oligopeptides **4** were prepared in high yields (70–97%), except for pS356 and pS352. The Acm group in **4** was removed using either PdCl_2_/MgCl_2_ or AgOAc in AcOH/H_2_O, producing unprotected peptides in 39–95% yield.^51,52^ The last peptide union was performed by a ligation between the free N-terminal cysteine and **3** using MPAA as the catalyst, and the Thz group was removed with MeONH_2_.^40,53^ All of the Tau(291–391) peptides behaved similarly under these conditions and produced comparable yields (Figure 2b). However, the Tau(291–322)–MeNbz fragment for the P301S mutant was poorly soluble in 6 M Gnd·HCl, and this fragment was solubilized with 8 M Gnd·HCl. The final peptides were purified by sample displacement mode chromatography with good yields achieved after LC.^54^ All of the proteoforms were homogeneous by LC-HRMS and stable to the subsequent manipulations (Figure 2c).

### B. Characterization of Tau(291–391)

**Figure 3:**
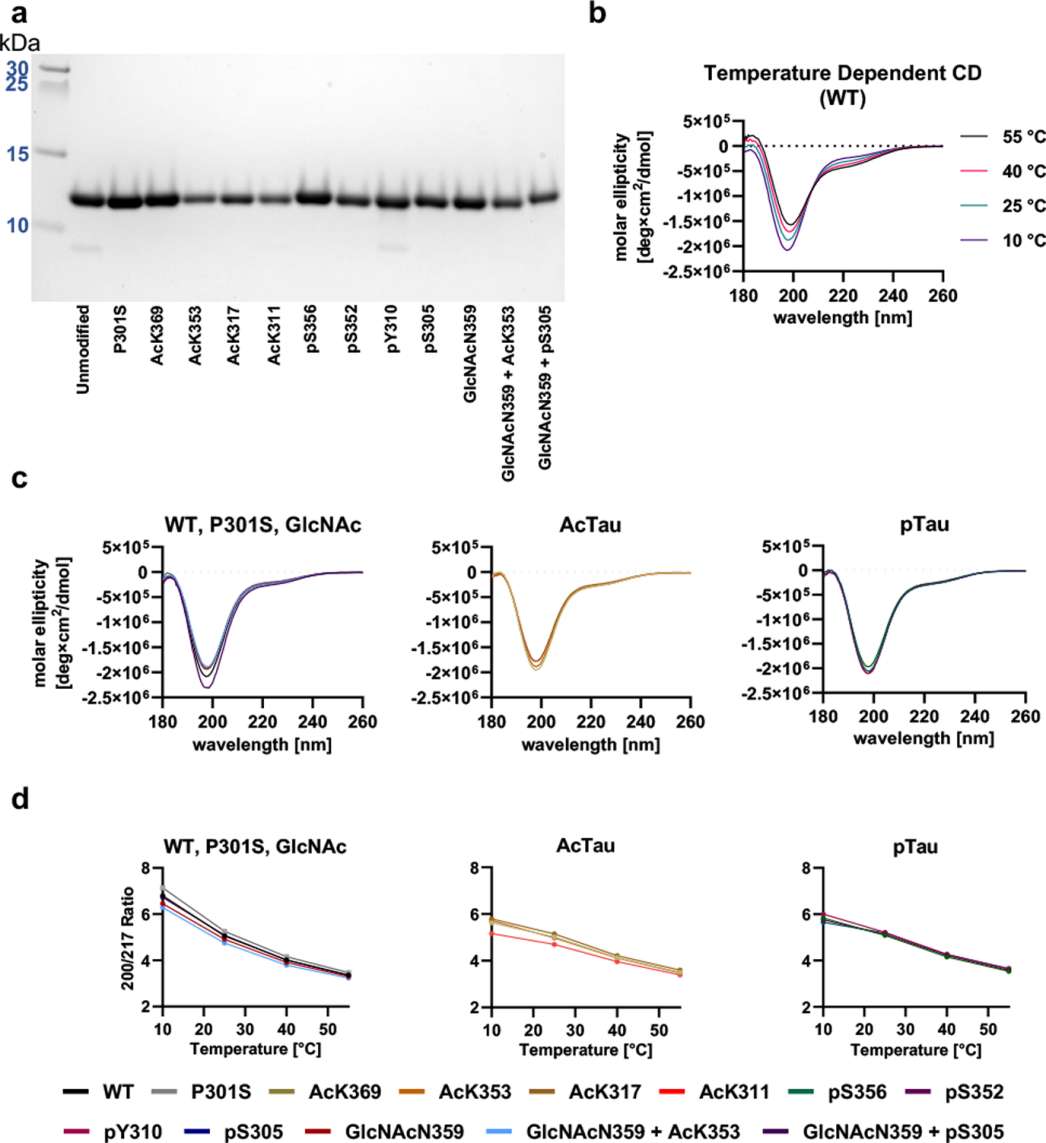
Characterization of the Tau(291–391) proteoforms. The CD experiments were performed in triplicate. (a) 16% SDS-PAGE (Novex Tricine) of the Tau(291–391) constructs with PTMs. (b) Temperature-dependent CD spectra of 0.4 mg/mL Tau(291–391) WT at 10, 25, 40, and 55 °C in 20 mM sodium phosphate buffer (pH 7.4). (c) CD spectra of 0.4 mg/mL Tau(291–391) at 25 °C in 20 mM sodium phosphate buffer (pH 7.4). (d) The ratio of θ at 200/217 nm for the Tau(291–391) constructs.

We characterized the synthetic Tau(291–391) proteins with gel electrophoresis to probe their gel mobility (Figure 3a). Abnormal Tau from the AD brain has a visible gel shift when compared to normal Tau due to the presence of PTMs.^55,56^ We found that with the exception of GlcNAcN359 + pS305, which has a slightly increased gel shift, the electrophoretic mobilities of all Tau(291–391) constructs were similar (all proteoforms have an apparent molecular weight of ∼12 kDa). From this study, we conclude that site-specific PTMs in this region of Tau do not cause a significant upward shift in the gel, supporting the observation that PTMs are present within proteolytically stable Tau PHFs from AD brains.^1,2^ To test if site-specific PTMs induce a conformational change in Tau, we used temperature-dependent circular dichroism (CD) spectroscopy (Figure 3b). The CD spectra of all Tau(291–391) proteoforms have a minimum around 200 nm, which is indicative of random coil, and a negative patch at 217 nm, which is indicative of β-structure.^57,58^ A positive peak at 217 nm could also indicate the presence of a PPII helix. However, distinguishing PPII or β-structure may be difficult when working with proteins and not short peptides.^59,60^ Upon heating to 55 °C, the point at 200 nm becomes less pronounced, and the patch at 217 nm becomes more negative. Additionally, there is an isosbestic point at around 210 nm, and the minimum peak at 200 nm undergoes a red shift of about 2 nm (Figure 3b). All the Tau constructs have a CD spectrum similar to that of the unmodified WT Tau with some differences in the intensity at 200 and 217 nm at low temperatures for acetyl and phosphorylated forms (Figure 3c). To better understand how the folding changes in response to temperature, we plotted the θ values for all proteoforms at 200 and 217 nm as a function of temperature (Figure 3d). This ratio would appear sigmoidal if cooperative folding occurs and as a straight line if there was a structural change without cooperative folding.^58^ For the unmodified Tau(291–391), P301S, and GlcNAcN359, the plot shows an open curve (from ∼7 to 3) but does not appear to have a sigmoidal shape.^58^ In contrast, the acetyl and phosphate modified constructs have similar plots that decrease (6 to 3) with a more sigmoidal curve. The most striking differences were noted at 10 °C: the acetyl and phosphate PTMs had smaller ratios (∼5.5) than WT and P301S (∼7). Interestingly, glycans (∼6.5) also had larger ratios at 10 °C. For WT, P301S, and GlcNAc, this could indicate that there is an additional contribution of PPII at low temperature, which is consistent with previous findings for N-linked glycoproteins.^61^ Our findings also contrast with previous reports of phosphorylated Tau having more PPII than WT, as the peak at 217 nm would be expected to be more positive.^62,63^ Taken together, the acetyl and phosphopeptides have more β-structure (or less PPII) at 10 °C than the GlcNAc and unmodified peptides, and all of the graphs converged upon heating. The 200/217 ratios suggest that WT and glycans undergo a structural transition that does not occur in a cooperative fashion, but the acetyl and phosphorylated peptides have a higher folding tendency at low temperature (although still not cooperative) because of the charge neutralization or increased hydrophobicity.

### C. Cofactor-Free Self-Assembly

**Figure 4:**
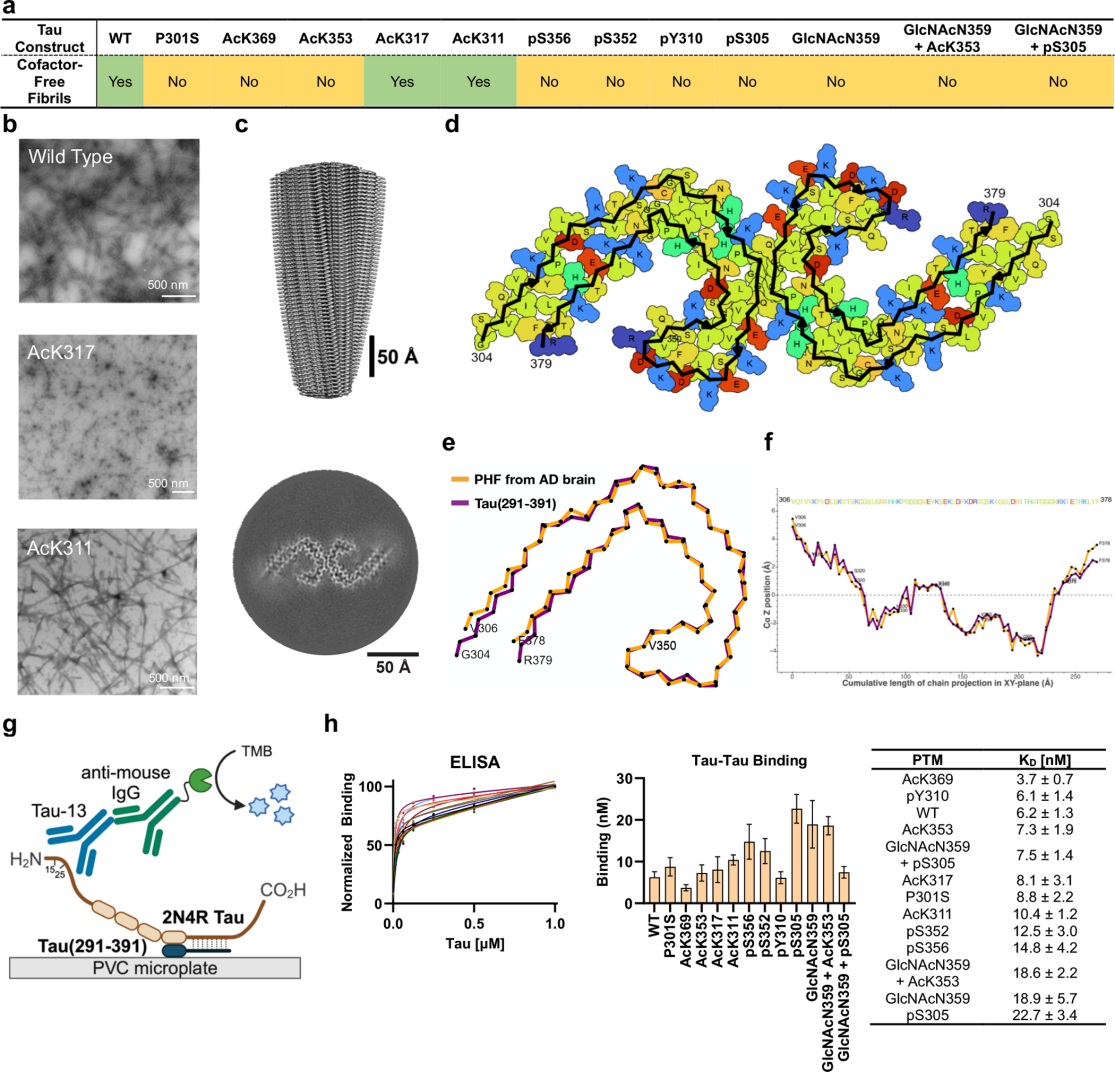
Cofactor-free self-assembly of Tau(291–391). (a) Summary of cofactor-free self-assembly of Tau(291–391) confirmed by negative stain TEM. Aggregation conditions: 4 mg/mL Tau(291–391), 200 mM MgCl_2_, 10 mM DTT, 20 mM Na_2_HPO_4_, pH 7.4, 37 °C, 48 h. All aggregation assays were performed in triplicate. (b) Representative TEM micrographs of Tau(291–391) proteoforms obtained under the standard conditions (three replicates, 10 pictures per replicate taken, and three pictures per replicate analyzed to determine the population distribution). (c, d) Cryo-EM structure of unmodified WT Tau(291–391). (e, f) Comparison between Tau(291–391) and PHFs from AD brain (PDB: 5O3L). (g) Schematic of ELISA to quantify interactions between Tau(291–391) and 2N4R Tau. In brief, Tau(291–391) proteoforms were immobilized on a PVC plate and treated with recombinant 2N4R Tau. Binding affinities were quantified colorimetrically (HRP) with Tau-13 mAb recognizing residues 15–25 of 2N4R Tau. Experiments conducted in the absence of 2N4R Tau in solution or the absence of immobilized Tau(291–391) resulted in minimal binding. (h) Langmuir binding curves and tabulated *K*_D_ values for ELISA. The apparent *K*_D_ values correspond to the average of three replicates, and the error bars represent SD.

Next, we tested the capacity of Tau constructs to self-assemble without cofactors into PHFs or other filamentous structures, hypothesizing that AD-specific post-translational modifications might enhance the formation of PHFs, making the self-assembly process more selective and potentially more efficient. We initiated the self-assembly of the constructs at a concentration of 4 mg/mL in MgCl_2_ buffer with 300 rpm shaking, a condition previously identified to facilitate PHF formation.^16^ Subsequently, the mixtures were negatively stained by TEM analysis. Three Tau constructs (WT, AcK317, and AcK311) were observed to form fibrils at a significantly high density (Figure 4a,b). The AcK317 samples displayed short and amorphous fibrils, whereas AcK311 yielded long rod-like filaments. The WT fibrils were considered suitable for cryo-electron microscopy (cryo-EM) analysis, leading to the generation of a high-resolution 3D structure resembling PHFs found in AD brains as well as recombinantly produced fibrils from Tau(297–391) (Figure 4c–f). However, attempts to produce high-resolution structures from the two acetyl derivatives were unsuccessful due to their fragile nature or their nontwisting topology. These findings suggest that (a) PTMs outside the ordered fibril core play a significant role in inducing Tau aggregation into PHFs, (b) the disease-specific PTMs we investigated are introduced post-PHF formation, targeting specific residues influenced by the pre-established conformation of the PHF, or (c) PTMs have low occupancy but high patient frequency.

The self-assembly of Tau(297–391) is facilitated by nanomolar Tau–Tau binding affinities.^64^ If the binding is primarily electrostatic, then PTMs that neutralize charge should enhance Tau–Tau binding by reducing the charge–charge repulsion. To evaluate this hypothesis, Tau(291–391) constructs were anchored on PVC microplates, and their interactions with 2N4R Tau were assessed using an N-terminal-specific monoclonal antibody, as shown in Figure 4g,h.^64,65^ ELISA is useful in comparing Tau–Tau interactions, binding information, and overall relative ranking between different proteoforms, although it cannot provide accurate *K*_D_ values by itself because one of the components is anchored to the solid phase and not in solution.

Except for pY310 and AcK369, each proteoform exhibited a reduced binding affinity to 2N4R Tau compared to the WT. If Tau–Tau binding is electrostatic, phosphorylated PTMs would show stronger binding due to enhanced charge neutralization. Nevertheless, our observations revealed that phosphorylated Tau demonstrated inferior binding compared with acetylated constructs, with pY310 being an exception. Proteoforms modified with GlcNAcN359, pS305, and the combination of GlcNAcN359 + AcK353 showed significantly diminished *K*_D_ compared to other PTMs, suggesting these modifications either introduce steric hindrance or destabilize crucial binding sites. Intriguingly, the combination of GlcNAcN359 + pS305 exhibited stronger binding than each modification individually, indicating that multiple PTMs with weak binding can synergize, leading to nonlinear effects on binding strength. Overall, our binding data imply that the Tau–Tau interaction may be governed more by hydrophobic and hydrogen bonding mechanisms rather than by electrostatic interactions, as charge-neutralizing PTMs appear to destabilize Tau–Tau binding.

### D. Aggregation Kinetics with Heparin

**Figure 5:**
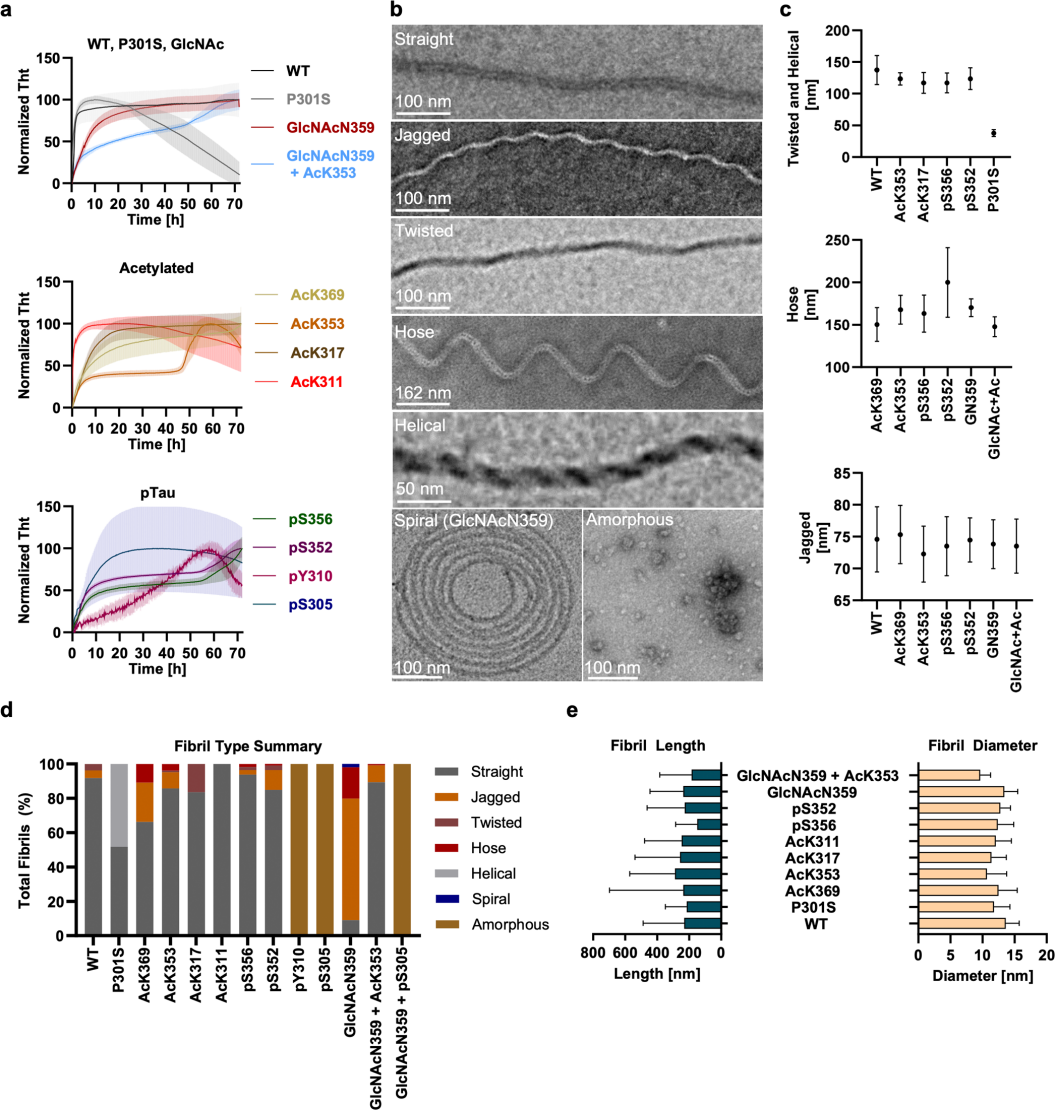
Heparin-induced aggregation of Tau(291–391). All experiments were performed in triplicate. (a) ThT aggregation kinetics of Tau(291–391) proteoforms in the presence of heparin. Aggregation assay conditions: 100 μM Tau(291–391), 40 μM heparin, 50 mM NaCl, 10 mM DTT, 10 μM ThT, 25 mM HEPES, pH 7.4, 37 °C, no shaking. The error bars represent SEM. (b) Representative negative stain images of various fibrils produced under conditions from Figure 5a. (c) Crossover distances of fibril morphology, measured by negative stain TEM, for proteoforms that form stable fibrils with heparin. The experiments were performed in triplicate, and three pictures from the three fibril batches were analyzed by FibrilJ (nine total images). The error bars represent SD. (d) Sorting of Tau(291–391) fibrils based on the general structural features depicted in Figure 5b. The experiments were performed in triplicate, and three pictures from the fibril batches were analyzed by FibrilJ (nine total images). (e) Length and diameter measurements of heparin-induced fibrils, determined with FibrilJ. The experiments were preformed in triplicate, and 10 pictures from the three fibril batches were analyzed by FibrilJ (30 total images). The error bars represent SD.

To better understand the role of PTMs in heparin-induced fibrillation, we examined the aggregation kinetics of various Tau(291–391) constructs. The kinetics were assessed based on the time required to reach half of the maximum Thioflavin T (ThT) fluorescence, a measure of amyloid formation kinetics, as well as the maximum ThT signal achieved (Figure 5a). The maximum ThT signal reached during the aggregation does not inform the final yield of the aggregates due to different intensities between variants and may also be due to how the fibril polymorphs and aggregates interact with ThT. Figure 5a shows that for all of the aggregation assays, the maximum ThT signal that was achieved was correlated with the kinetics because the proteoforms with faster kinetics have higher ThT signal. Furthermore, all the ThT signals have a similar order of magnitude, with the exception of that of pY310, which indicates that this proteoform results in minimal amounts of fibril aggregates. The structures modified with acetylation displayed three distinct kinetic profiles: WT and AcK311 exhibited rapid aggregation, achieving half-maximum signal within an hour; AcK317 demonstrated slower aggregation kinetics compared to WT; and both AcK353 and AcK369 also showed reduced kinetics similar to AcK317 but uniquely featured a second plateau, indicating a complex aggregation process. Phosphorylated constructs, in general, aggregated more slowly than the WT peptide. The pS356, pS352, and pY310 modifications all exhibited first and second kinetic plateaus akin to those of AcK353 and AcK369. Notably, pS305 and pY310, despite generating a ThT signal, failed to form filamentous structures under negative stain TEM, presenting only as amorphous aggregates. The GlcNAcN359 modification slightly decelerated the aggregation relative to WT, while the neutral P301S mutation expectedly sped up the process. The combined GlcNAcN359 + AcK353 modification mirrored the kinetics of AcK353 alone, with a slower aggregation rate and a secondary plateau. The GlcNAcN359 + pS305 construct, however, did not produce a significant ThT signal, and filamentous structures were absent in TEM analyses, highlighting the profound impact of specific PTMs on Tau aggregation pathways. Collectively, our findings show that PTMs generally reduce the heparin-induced aggregation kinetics of Tau, with some exceptions. Acetylation at specific sites, such as K311, accelerates aggregation, whereas acetylation at K369, K353, and K317 hinders the process, echoing similar findings for site-specific acetylation.^46,66−70^ Phosphorylation diminishes heparin-induced aggregation, aligning with prior studies.^45,71,72^ The GlcNAc modification slows aggregation and further contributes to our understanding of how *N*-glycosylation influences Tau pathology.^73^ Additionally, constructs with dual modifications tend to reflect the characteristics of their charge-neutralizing modifications. The P301S mutation’s pronounced effect in accelerating aggregation emphasizes the importance of genetic variations in tauopathy manifestations.^74,75^

### E. TEM Analysis of Fibril Types

Given the distinct reaction kinetics presented by various PTMs, we examined the morphology of heparin-induced fibrils using negative stain TEM. This analysis revealed heterogeneous fibril populations including helical, jagged, twisted, hose-like, and straight morphologies (Figure 5b,c). Notably, straight fibrils emerged as the predominant form across the samples (Figure 5d). However, the GlcNAcN359 modification predominantly yielded jagged fibrils, while P301S was unique in forming a significant proportion of helical structures. Jagged fibrils were also common, with WT, AcK369, AcK353, pS356, pS352, and GlcNAcN359 + AcK353 samples exhibiting 5–10% of this type. Hose-like fibrils were observed in several proteoforms including AcK369, AcK353, pS352, pS356, and GlcNAc, while twisted fibrils were identified in WT, AcK317, and pS352 samples, albeit in smaller proportions. This analysis concluded that PTMs significantly influence the distribution of fibril morphologies in heparin-induced aggregates, with certain modifications exerting dominant effects and those in proximity showing similar morphological distributions.

In examining the crossover distances of the various fibril types, we found uniformity across the morphologies, with each type displaying consistent crossover distances indicative of structural similarities (Figure 5c). The consistent crossover distances among different fibril types further imply that despite the varied morphologies, there is a structural resemblance within each type.^12^ This comprehensive analysis concludes that PTMs significantly influence the distribution of fibril morphologies in heparin-induced aggregates, with certain modifications exerting dominant effects and those in proximity showing similar morphological distributions.

Further analysis with FibrilJ underscored small variations in fibril diameters and lengths (Figure 5e). Acetylation consistently reduced the average fibril diameter by 1–3 nm, whereas glycosylation alone had a lesser impact. Interestingly, phosphorylation mirrored the diameter reductions seen with acetylation. Notably, the P301S modification led to a broader diameter distribution, with an average reduction of 1.5 nm compared to WT. Kolmogorov–Smirnov tests confirmed the statistical significance (*p* < 0.0001) of these diameter variations, highlighting the impactful role of PTMs and mutations on fibril width distribution.

Fibril length analysis revealed that most modifications, except for AcK353, did not produce fibrils exceeding the lengths observed in WT samples. P301S fibrils exhibited the most constrained length distribution, with the majority measuring between 100 and 200 nm. This contrast in fibril lengths was also statistically significant across all groups (*p* < 0.0001), suggesting that while modifications influence fibril morphology and diameter, their impact on length is less consistent.

### F. RNA-Induced Liquid–Liquid Phase Separation

**Figure 6:**
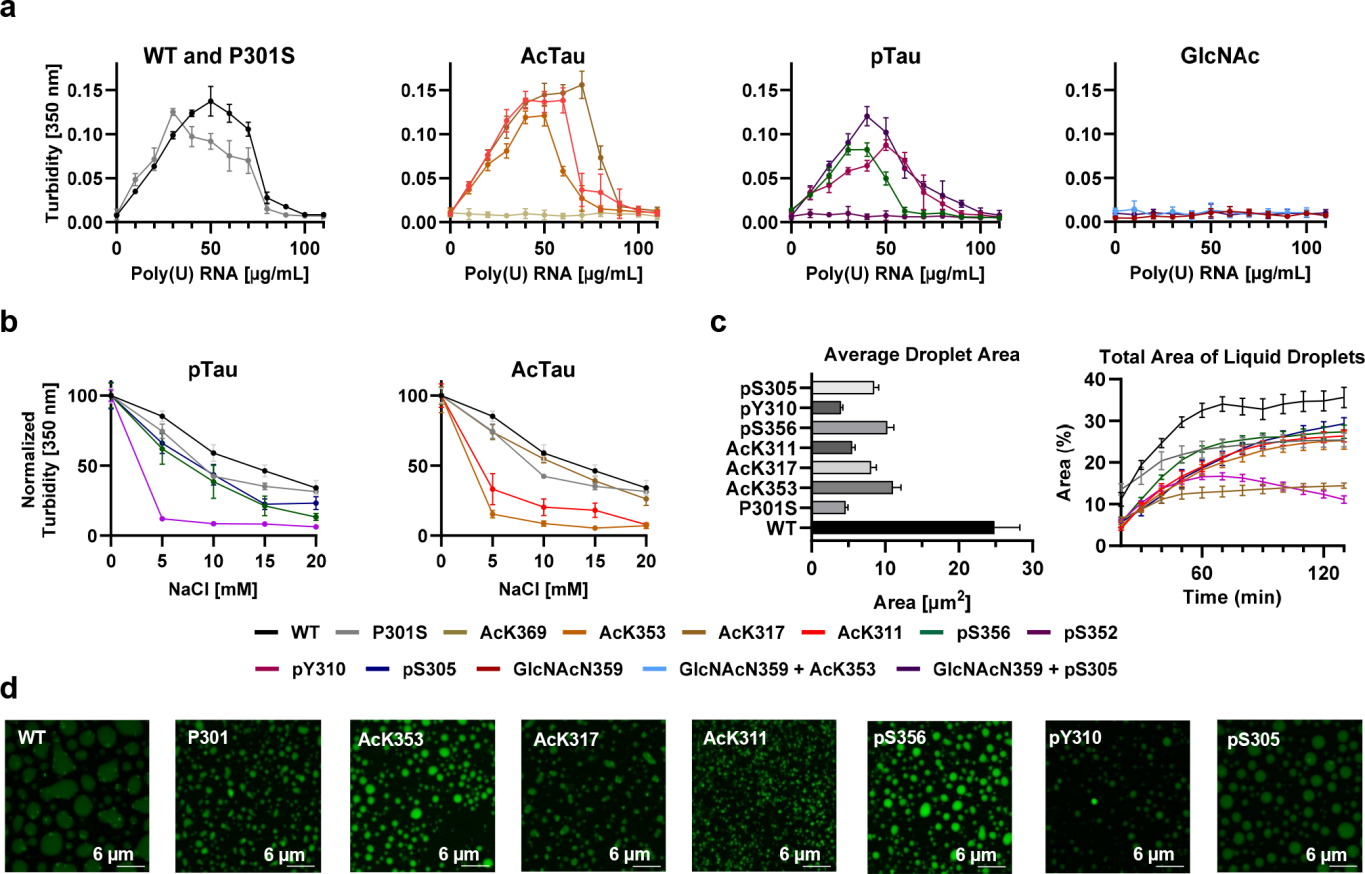
RNA-induced liquid–liquid phase separation of Tau(291–391). The turbidity and microscopy experiments were performed in triplicate, and the error bars represent SEM. (a) Phase diagram of poly(U) RNA-induced droplets. Turbidity was measured at 350 nm, 2 min after mixing with 30 μM Tau(291–391), variable μg/mL poly(U) RNA, 10 mM DTT, 25 mM HEPES, pH 7.4, 25 °C. (b) Salt resistance in response to varying NaCl concentration measured by absorbance at 350 nm. Salt resistance assay diagram with 30 μM Tau(291–391), 40 μg/mL poly(U) RNA, 10 mM DTT, 25 mM HEPES, pH 7.4, 25 °C, and variable mM NaCl. (c) Average droplet area 2 h after preparing the droplets (left) and the total area of droplets that cover the microscope slide over time (right). (d) Images of Tau(291–391)–poly(U) RNA droplets, after 2 h, with 30 μM Tau(291–391), 0.3 μM AF488 (adjusted to 1% labeling with AF488 labeled Tau(291–391)), 40 μg/mL poly(U) RNA, 10 mM DTT, 25 mM HEPES, pH 7.4, 25 °C. Scale bar = 10 μm^2^.

LLPS of Tau may be an important intermediate phase that helps in the nucleation or oligomerization of Tau before fibrils are fully formed.^76^ This phase transition may also aid in concentrating Tau from a dilute solution to a more concentrated phase, where aggregation occurs. Therefore, we wanted to know whether LLPS of Tau(291–391) is a relevant phase transformation that aids in the aggregation of Tau. Like amyloid aggregation, there are two types of LLPS mechanisms: complex coacervation and self-coacervation. Electrostatically driven complex coacervation of Tau occurs when the positively charged lysine and arginine residues of Tau bind to negatively charged RNA or other types of polyanions such as ssDNA, heparin, hyaluronic acid, and α-synuclein.^77−81^ Full-length Tau also undergoes electrostatically driven self-coacervation upon the addition of molecular crowding agents (PEG, dextran) through attractive intramolecular electrostatic interactions between the negatively charged N-terminus and the positively charged MTBR.^82−85^ Hydrophobically driven self-coacervation of Tau occurs under high salt conditions or hyperphosphorylation, which weaken electrostatic interactions.^86,87^ However, Tau(291–391) did not undergo *in vitro* self-coacervation, as no droplets were observed even at 500 μM Tau(291–391) under molecular crowding (15% PEG-10) or high salt (4.75 M NaCl) conditions. Nevertheless, Tau(291–391) underwent complex coacervation with RNA: treatment of Tau(291–391) with poly(U) immediately formed a visibly turbid solution, and liquid droplets were observed by fluorescence microscopy (Figure 6).

Next, we wanted to test the effect of PTMs on the phase diagram of Tau(291–391). LLPS with RNA has a peak turbidity when the two components are under charge matching conditions, and the turbidity values decrease when the positive and negative charge concentrations are unequal until only a singular phase system exists.^79,88^ The intensity of turbidity values is semiquantitative and can depend on the shape, size, and density of the droplets. However, the range of RNA concentrations and the RNA concentration at maximum turbidity provide valuable insights.^89^ Tau(291–391) contains 15 lysine and 2 arginine residues, which undergo multivalent binding with the phosphates of poly(U), driving complex coacervation.^78^ We expected that P301S and *N*-glycosylation, two neutral PTMs, would have no effect on LLPS. In contrast, acetylation and phosphorylation were expected to alter the peak turbidity and decrease the range of RNA concentrations for LLPS because they (a) decrease the number of binding sites (acetylation) or weaken the interaction strength (both phosphorylation and acetylation), (b) lower the isoelectric point, and (c) have less net positive charge.^78,88,90^

To validate these hypotheses, we generated a phase diagram of Tau with poly(U) (Figure 6a). We titrated a fixed concentration of Tau (30 μM) with poly(U) ranging from 0 to 110 μg/mL and measured absorbance at 350 nm as an indicator of LLPS. The phase diagram revealed that WT Tau(291–391) has a maximum turbidity value around 50 μg/mL RNA, and it spans the range of 0–100 μg/mL poly(U). Acetylation at K311 and K317 has only small effects on the phase diagram compared to the WT protein. However, AcK353 has a reduced two-phase window, and AcK369 completely suppressed LLPS. Similar to our findings on monoacetylation at AcK311 and AcK317, monoubiquitination at K311 and K317 was reported to have minor effects on K18 LLPS with poly(U), based on droplet size vs time analysis.^91^ Phosphorylation of Y310 is similar to WT, while S305 and S356 shift the peak turbidity value to 40 and 30 μg/mL and narrow the phase diagram; pS352 suppresses LLPS. Single *N*-glycosylation (GlcNAc) completely suppressed complex coacervation. Furthermore, the addition of AcK353 or pS305 on the same peptide chain also does not rescue the droplet formation ability. The P301S mutation displays a similar two-phase window as the unmodified peptide; however, the peak turbidity value is shifted to 30 μg/mL poly(U). In conclusion, our findings demonstrate that phosphorylation reduces LLPS, while acetylation can have a positive or negative effect. This implies that certain residues are crucial for RNA binding and PTMs can impair these LLPS interaction sites. While some PTMs, such as AcK369, pS352, and GlcNAcN359, negatively affect RNA binding, others show moderate or no significant impact on the phase diagram.

### G. Salt Resistance

Tau displays a decreased tendency to undergo LLPS at increasing salt concentrations because the salt competes for the electrostatic attraction between ammonium or guanidinium sites to the phosphate groups of RNA.^77,79,84,92,93^ We hypothesized that PTMs exacerbate site-specific effects related to denaturation with salt, and some PTMs, even with the same charge, may exhibit unequal LLPS propensities. The turbidity of WT and 40 μg/mL poly(U) decreased by half in the presence of 20 mM NaCl, and a similar trend occurred for P301S and AcK317 (even though one of the lysine residues is unavailable for binding with RNA; Figure 6b). Droplets produced from AcK311 and AcK353 were less stable toward salt, and the turbidity was diminished at 20 and 15 mM NaCl, respectively. Phosphorylation at S356 and S305 leads to less salt resistance than the WT peptide, and turbidity diminished to ∼25% at 20 mM NaCl. Among all the proteoforms studied, pY310 was the least salt-resistant, and LLPS was diminished at 10 mM NaCl. These data suggest that PTMs have differential effects on the salt resistance of LLPS of Tau, and some regions are more important for maintaining the charge in high salt solutions. The droplets of AcK311 and AcK353 diminished quickly, suggesting that these residues may play a crucial role in maintaining RNA binding. The pY310 proteoform has a similar phase diagram as WT, but LLPS is rapidly diminished with salt, which shows the unique effect of this phosphorylated residue together with its aromatic character.

### H. Analysis of Droplet Size over Time in Relation to PTMs

Our investigations also focused on examining how PTMs influence the morphology and dynamics of liquid phase-separated droplets associated with Tau. By employing time-lapse microscopy, we were able to track changes in the total area covered by these droplets over time, with the results illustrated in Figure 6c. The droplets demonstrated liquid-like properties, including droplet fusion and Ostwald ripening, and the area they covered on the microscopy slide plateaued after 1 h. Interestingly, the coverage area was consistent across most constructs, with the notable exceptions being WT, which covered a larger area, and the AcK317 and pY310 constructs, which covered a smaller area. This suggests variations in the efficiency of monomer to droplet conversion or differences in droplet density, with WT droplets being less dense than those formed by AcK317 and pY310. By the 2 h mark, the droplets began to exhibit distinct morphologies: AcK353, pS305, pS356, and pY310 predominantly formed circular droplets; WT and P301S showed a mixture of circular and amorphous droplets; and AcK317 and AcK311 mostly produced smaller, amorphous droplets, as depicted in Figure 6d. Further analysis at this time point focused on the average area of the droplets (Figure 6c), revealing that WT droplets were the largest, with an average size of 35 μm^2^, while AcK317 and pY310 formed the smallest droplets, averaging 15 μm^2^. Droplets from the other constructs had an intermediate average size of about 25 μm^2^. These observations underscore the significant role of PTMs in modulating both the morphology and the apparent density of the Tau liquid droplets. Regardless of their shape, be it circular or amorphous, all droplets retained a liquid-like appearance. Their size steadily increased until a stable state was achieved approximately 2 h into the observation period, as the droplets settled on the slide surface. An anomaly was noted with the pY310 droplets, which exhibited a tendency to compress rather than settle, highlighting the unique behaviors induced by the specific PTMs.

### I. Droplet Dynamics

We next explored whether amyloid fibril nucleation occurs during the aging of Tau(291–391)–RNA droplets and how PTMs might differentially affect this process. Liquid phase-separated droplets of intrinsically disordered proteins can undergo aging into gel-like structures, Maxwell glasses, or amyloid fibrils.^76,79,94^ However, the specific trajectory of Tau(291–391) during LLPS with RNA, particularly whether it undergoes fibrillation, is unknown. Pathogenic Tau mutations are known to facilitate the aging of liquid droplets into higher molecular weight species, but amyloid fibril nucleation typically requires polyanionic cofactors.^82,85,86,95^ Conversely, LLPS driven by hydrophobic interactions, such as those induced by high salt conditions or hyperphosphorylation, is known to trigger amyloid fibril formation.^86,87^

**Figure 7:**
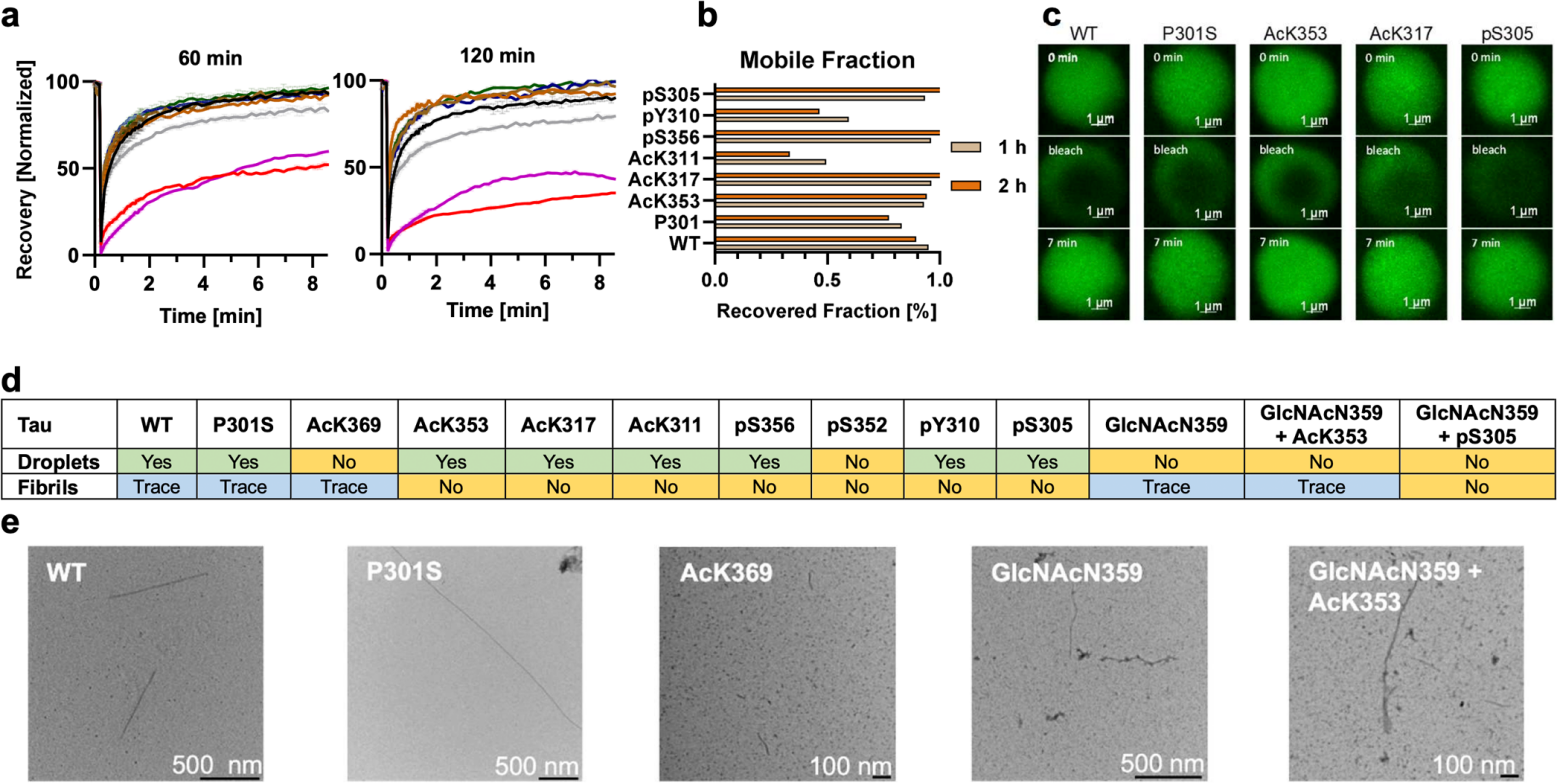
RNA-induced liquid–liquid phase separation of Tau(291–391). (a) FRAP kinetics for Tau(291–391) constructs with PTMs after 1 and 2 h: 30 μM Tau(291–391), 0.3 μM AF488, 40 μg/mL poly(U) RNA, 10 mM DTT, 25 mM HEPES, pH 7.4, 23 °C. (b) Calculated mobile fraction of the droplets 1 and 2 h after preparing the droplets. (c) Representative images before, during, and after photobleaching, taken 1 h after preparing the droplets. (d) Summary of droplet and fibril formation under LLPS conditions: 30 μM Tau(291–391), 40 μg/mL poly(U) RNA, 10 mM DTT, 25 mM HEPES, pH 7.4, and 23 °C. The droplets were detected by turbidity after 2 h, and the fibrils were detected by negative stain TEM after 3 days. (e) Negative stain images of fibrils formed under LLPS conditions after 3 days.

To investigate these aspects of LLPS, we employed fluorescence recovery after photobleaching (FRAP) to probe the liquid–solid transition during complex coacervation.^96^ Additionally, we used negative stain TEM to check for the presence of amyloid fibrils (Figure 7a–e). FRAP assessments conducted 1 h into the experiment showed that Tau–RNA droplets from WT, P301S, AcK353, AcK317, pS356, and pS305 predominantly maintained their liquid state, demonstrating near-complete fluorescence recovery. Conversely, droplets from AcK311 and pY310 exhibited diminished dynamics, as reflected by a reduced mobile fraction (Figure 7b). After 2 h, a decrease in mobility was noted for WT, P301S, pY310, and AcK311 droplets, suggesting gradual solidification. Nonetheless, droplets from AcK353, AcK317, pS305, and pS356 retained a similar mobility, suggesting persistent liquidity. Further analysis using negative stain TEM after 2 h revealed no fibril formation at this stage (Figure 7d,e). However, sparse fibril density was observed in several constructs after 3 days, including WT, P301S, AcK369, GlcNAcN359, and GlcNAcN359 + AcK353. This suggests that while WT, P301S, AcK311, and pY310 droplets show a decrease in mobility over time, indicative of gelation or Maxwell glass formation, this does not necessarily lead to amyloid fibril formation, as no fibrils were visible after 3 h. This observation underscores the notion that droplet formation and fibrillation are distinct processes, offering insights into Tau behavior during phase transitions and the complex interplay between LLPS and protein aggregation.^79^

## Discussion

In this study, we explored the mechanisms behind Tau filament formation by examining the behavior of Tau protein segments and their interaction with post-translational modifications. The Tau protein in AD brains exists in three distinct forms: a normal cytosolic form, a soluble hyperphosphorylated form, and an insoluble paired helical filament form.^55^ Furthermore, the Tau from AD patients is also known to contain other post-translational modifications, and it is not understood how PTMs regulate the (*in vitro*) formation of AD filaments or which phase transformations are relevant for PHF formation on full-length Tau. The paired helical filaments from AD patients consist of a rigid core surrounded by a “fuzzy coat”, which can be removed to reveal the proteolytically stable segment Tau(297–391).^1,2^ This finding is critical, as it suggests a specific region of Tau (I297–E391 and L266–E391, corresponding to 4R and 3R Tau, respectively) is capable of maintaining its assembly without any cofactors.^97−101^ The morphology of these self-assembled filaments closely resembles that of those extracted from brain tissue, as demonstrated by negative stain TEM and atomic force microscopy.^102−105^ The observation that Tau(297–391) can self-assemble into filaments that matches the ones isolated from AD and CTE patients was further confirmed with cryo-EM; however, the structures are largely dependent on the shaking speed and buffer composition.^16,18^ To investigate the role of PTMs in filament formation, we developed a three-segment chemical synthesis of Tau(291–391), which is a promising region for self-assembly. This approach allowed us to produce 13 unique proteoforms with site-specific PTMs, including phosphorylation, acetylation, *N*-glycosylation, and a point mutation (Figures 1 and 2). The solution-phase behaviors of these proteoforms were largely identical. However, significant differences were observed, as the proteoforms were tested under various phase transition conditions. Tau fibril formation involves a liquid to solid phase transition known as nucleation, followed by secondary processes that catalyze further fibril nucleation.^106^ Although Tau aggregation can be accomplished under cofactor-induced and cofactor-free conditions, the exact phase transitions and the role of oligomeric species or liquid phase-separated condensates remain complex and not fully understood.^107−111^ The essential role of polyanionic cofactors in the formation, stabilization, and aggregation kinetics of polyanion-induced filaments has been established,^112−116^ yet *in vitro* filament preparations with polyanions do not replicate the cryo-EM structures of filaments from human patients.^12,13^ Our findings emphasize the complexity of Tau aggregation and the significant impact of PTMs on filament formation. The WT, AcK311, and AcK317 constructs demonstrated self-assembly, but only WT fibrils formed PHF filaments under previously optimized conditions (confirmed through a cryo-EM structure that matched those isolated from AD brains).^16,18^ The self-assembly is facilitated by Tau–Tau binding, which exhibits nanomolar affinities and is correlated with positional specificity and PTM identity.^117^ Interestingly, the AcK311 and P301S mutations accelerated heparin-induced aggregation, whereas AcK353, AcK369, AcK317, pS356, pS352, GlcNAcN359, and GlcNAcN359 + AcK353 inhibited aggregation. Moreover, pS305 and pY310 mutations completely suppressed self-assembly with heparin, underscoring the critical role of specific PTMs in regulating Tau filament formation.

LLPS may also be an important intermediate phase that concentrates Tau from the dilute solution and enables nucleation or oligomerization. Phase separation is related to aggregation, as it occurs under both polyanion-induced and cofactor-free conditions.^77,78,82−88^ We established that Tau(291–391) undergoes RNA-induced liquid–liquid phase separation, and the AcK369, pS352, and GlcNAcN359 modifications completely suppressed LLPS. RNA-induced LLPS does not help promote the fibrilization of Tau(297–391) under the conditions tested with poly(U) RNA (Figure 7); rather, gelation occurs during this step, and RNA-promoted aggregation is a separate process with the truncated construct. It remains under debate whether Tau phase separation can drive the formation of fibrils and oligomers, or if it is an off pathway phase transformation that occurs under similar conditions.^79,85,86^ Our work indicates that this process may be controlled by specific PTMs, as the pathway for the aggregation or unproductive LLPS is dependent on the PTM identity and even position on Tau. These observations add to the complexity of the mechanism of self-assembly into PHFs.

The strength of our study lies in its ability to investigate the effects of site-specific PTMs on Tau aggregation. We have demonstrated that the specific residue carrying a post-translational modification can influence Tau aggregation positively, as evidenced by AcK311. Conversely, the same type of modification at a different site, such as AcK369, can have a negative impact. This finding underscores the complex yet significant role of PTMs in Tau aggregation. One interpretation of these data is that if the PTMs are on the full-length protein, the PTMs within the ordered filament core may aid in the self-assembly of full-length hyperphosphorylated Tau. An alternative interpretation is that AD-specific PTMs within the ordered filament core are present on insoluble Tau PHFs because they are at conformationally accessible residues and become modified because of reduced Tau clearance.

A limitation of our approach is the use of a Tau fragment rather than a full-length protein, which is more representative of clinical conditions. Notably, several PTMs are located within the ordered filament core in AD patients, but many are also found outside this region.^33^ Furthermore, our attempts to study the interplay of two PTMs represent only a fraction of possible (and likely) combinations of PTMs in an individual Tau chain. In the disease state, the filaments are heavily modified with PTMs,^33^ and future studies on Tau constructs that contain higher modification stoichiometry may provide a more comprehensive analysis of the interplay between the PTMs.

In conclusion, our work has advanced the understanding of Tau protein aggregation by demonstrating that site-specific PTMs can have varying effects on the aggregation process, with some facilitating and others inhibiting the formation of PHFs. These insights into RNA-induced LLPS indicate that it does not contribute to the nucleation of AD filaments, emphasizing the role of environmental factors and PTMs located outside the ordered filament core in Tau self-assembly. Our findings lay the groundwork for future studies on full-length and hyperphosphorylated Tau constructs and the influence of higher modification stoichiometries on Tau aggregation, which could further elucidate the pathogenesis of Alzheimer’s disease and related tauopathies.

## Methods

### Peptide Synthesis and Purification

Peptide synthesis was accomplished with standard Fmoc-based chemistries using automated peptide synthesizers (room temperature or microwave-assisted coupling). The crude peptides obtained after resin cleavage were purified by preparative HPLC and lyophilized. The identity and purity of the peptides were confirmed by LC-HRMS.

### CD Spectroscopy

Lyophilized Tau(291–391) was dissolved in 20 mM sodium phosphate buffer (pH 7.4), and the residual TFA salts were removed using a 7000 MWCO Slide-A-Lyzer MINI Dialysis Unit (Thermo Scientific, cat. no. 69562). The proteins were adjusted to 0.4 mg/mL in 20 mM sodium phosphate buffer (pH 7.4) and analyzed in triplicate with CD spectroscopy. The triplicate CD data were averaged and reported as the molar ellipticity.

### Cofactor-Free Self-Assembly

This protocol was adapted from the literature.^16^ Lyophilized Tau(291–391) was dissolved in 10 mM sodium phosphate and 10 mM DTT (pH 7.4) and purified by SEC over a Superdex 75 10/300 GL into 10 mM sodium phosphate and 10 mM DTT (pH 7.4). The fractions containing protein were concentrated to 8 mg/mL using a Pierce concentrator (PES, 3K MWCO, 0.5 mL, 88512).

The cofactor-free self-assembly was run in a 96-well plate (Greiner BioOne, 96-well, PS, F Bottom, Chimney well, black, medium binding, 655096). The wells were filled with 100 μL of the sample with 4 mg/mL Tau(291–391), 200 mM MgCl_2_ (using a pH 7.4 stock solution of 0.8 M MgCl_2_ in 10 mM sodium phosphate), 10 mM DTT, and 10 mM sodium phosphate (pH 7.4). The plate was sealed with an adhesive film (VWR, polyester foil, 89134-430) and mixed at 37 °C at 300 rpm on a thermomixer (Ika Matrix Orbital). After 48 h, the aggregation mixtures were directly assayed for fibrils by negative stain TEM.

### Heparin Protein Aggregation

A 0.65 μL Eppendorf tube was charged with 50 μL of aggregation assay mixture at the final concentration listed. The buffers and salts were added first, followed by protein, and heparin was added last. Three wells on a black, 384-well, nonbinding microplate (Greiner BioOne, PS, F Bottom, small volume, HiBase, 784900) were filled with 15 μL of the aggregation assay mixture. The plate was sealed with polyester adhesive film (VWR, 89134-430), and the plate was incubated at 37 °C into the microplate reader, monitoring the fluorescence (Ex/Em: 440/480 nm). The data from the three wells were averaged, and the experiment was repeated three times. The data were averaged and then normalized, and the error bars on the graphs are reported as the standard error of the mean.

### ELISA

This protocol was taken from the literature with minor modifications.^117,118^ The difference with our protocol is that we used 2N4R Tau(2–441) in the solution phase instead of Tau(297–391), and we used anti-Tau, 15–25 mouse antibody (binds residues 15–25 on 2N4R Tau, BioLegend, 835201) as the primary antibody instead of mAb 423 and anti-mouse IgG (H+L), HRP conjugate (Promega, W4021) as the secondary antibody.

### Phase Diagram and FRAP

Turbidity phase diagrams were measured on a NanoDrop2000 instrument. The absorbance was measured at 350 nm with a path length of 0.1 cm. A 0.65 μL Eppendorf tube was charged with 3 μL of 2× protein stock. Next, 3 μL of 2× poly(U) RNA stock solution was added, and the mixture was pipetted up and down several times. A minute after mixing, 2 μL of the sample was transferred to the NanoDrop instrument, and the absorbance was measured three times. Using the same LLPS mixture, this was repeated twice. This experiment was performed in triplicate, and the data from the three experiments were averaged and reported with error bars that represent the standard error of the mean.

### Salt Resistance

The salt resistance assay was measured by turbidity on a NanoDrop2000 instrument. The absorbance was measured at 350 nm with a path length of 0.1 cm. A 0.65 μL Eppendorf tube was charged with 3 μL of 3× protein stock. The mixture was treated with 1.5 μL of 6× NaCl stock. Next, 4.5 μL of 2× RNA stock was added, and the mixture was pipetted up and down several times. A minute after mixing, 2 μL of the droplet solution was transferred to the NanoDrop instrument, and the absorbance was measured three times. Using the same LLPS mixture, this was repeated twice. This experiment was performed in triplicate, and the data from the three experiments were averaged and reported with error bars that represent the standard error of the mean.

### Droplet Size Measurements by Fluorescence Microscopy

A 0.65 μL Eppendorf tube was charged with 3 μL of 2× protein. Next, 3 μL of 2× poly(U) RNA stock was added, and the mixture was pipetted up and down several times. The sample was transferred to the center of the microwell in a 35 mm glass bottom dish with a 14 mm microwell. The edge of the microwell was lined with 25 mM HEPES (∼12 μL) to provide an evaporation shield; the microwell was covered with a glass coverslip, and the chamber was sealed with nail polish. For each droplet preparation, five random locations were imaged in 10 min intervals. The experiment was repeated for a total of three or four independent experiments.

### FRAP

A 0.65 μL Eppendorf tube was charged with 3 μL of 2× protein stock. Next, 3 μL of 2× poly(U) RNA stock was added, and the mixture was pipetted up and down several times. The sample was transferred to the center of the microwell in a 35 mm glass bottom dish with a 14 mm microwell. The edge of the microwell was lined with 25 mM HEPES (∼12 μL) to provide an evaporation shield. The microwell was covered with a glass coverslip, and the chamber was sealed with nail polish. For each droplet preparation, one droplet is photobleached after 1 h, and another droplet was photobleached after 2 h. The experiment was repeated for a total of five independent experiments.

### Assembly of 291–391 (Cryo-EM)

Lyophilized samples were resuspended in 1 mL of 10 mM PB pH 7.4 and 10 mM DTT and left for 20 min at RT. Samples were then centrifuged (13 000 rpm for 5 min at 20 °C) prior to size exclusion chromatography (HiLoad Superdex 200 pg, Cytivia**)**. Fractions containing protein were verified by SDS-PAGE (4–20% Tris Glycine) and pooled and concentrated to 8 mg/mL using molecular weight concentrators with a cutoff filter of 3 kDa. Protein concentrations were determined using a NanoDrop2000 instrument (Thermo Fisher Scientific). 291–391 was diluted to 5 mg/mL containing 10 mM phosphate buffer at pH 7.2, 100 mM MgCl_2_, and 10 mM DTT and assembled in a 384-well microplate that was sealed and placed in a Fluostar Omega instrument (BMB Labtech) with 200 rpm shaking at 37 °C for 24 h.

### Cryo-EM

After 24 h, the sample was taken directly from the microplate, and 3 μL of the reaction mixture was applied to glow-discharged R1.2/1.3, 300 mesh carbon Au grids. The grids were plunge-frozen in liquid ethane using a Vitrobot Mark IV instrument (Thermo Fisher Scientific). Cryo-EM images were acquired on a Krios G2 (Thermo Fisher Scientific) electron microscope that was equipped with a Falcon-4 camera (Thermo Fisher Scientific). Images were recorded at a dose of 30 electrons per square ångström using EPU software (Thermo Fisher Scientific) and converted to tiff format using relion_convert_to_tiff.

### Cryo-EM Data Processing

Movie frames were gain corrected, aligned, and dose weighted using RELION’s motion correction program. Contrast transfer function (CTF) parameters were estimated by using CTFFIND-4.1. RELION helical reconstruction was carried out using RELION-4.0. Filaments were picked manually and extracted in a box size of 768 pixels down-sampled to 128 pixels for the initial 2D classification. Good classes were selected and re-extracted in a particle box size of 384 pixels with no down-sampling for 3D auto-refinements. After several rounds of 3D auto-refinement optimizing for helical parameters, Bayesian polishing and CTF refinement were used to further increase the resolution. Final maps were sharpened using standard postprocessing procedures in RELION. Reported resolutions were estimated using a threshold of 0.143 in the Fourier shell correlation (FSC) between two independently refined half-maps.

### Negative Stain TEM of Tau Filaments

Filaments were negatively stained with 3% uranyl acetate and imaged on an FEI Tecnai T12 Spirit instrument at 120 kV with a LaB_6_ filament. Three replicates of each group were imaged, and 10 random images at 30 000× (2.119 nm/pixel) were obtained per replicate. Data from these replicates were compared and then pooled per group for subsequent analysis by FibrilJ.

### Analysis of the Fibrillar Length and Diameter

Tau(291–391) fibrils were analyzed using the ImageJ fibril analysis plugin, FibrilJ.^119^ A modified version of FibrilJ, incorporating an enhanced skeleton pruning algorithm, was employed to streamline the fibrils into lines and reduce the occurrence of branches that were erroneously generated during skeletonization. Before analysis, images underwent binarization via custom segmentation algorithms. Among the three diameter algorithms provided by FibrilJ, results from the D2 (human-like) algorithm are highlighted in the Results section, as the D1 (area/length) and D3 (distance map) algorithms yielded inconsistent calculations. FibrilJ’s ability to accurately determine fibril lengths is constrained by the inherent limitations of the ImageJ 2D/3D Skeleton program and the AnalyzeSkeletons-based pruning plugin. In the pruning process, FibrilJ trims the shortest branches, sometimes inadvertently dividing fibrils at arbitrary points along their length. This issue is particularly prevalent when fibrils intersect or overlap or when staining is uneven, affecting the binary image quality. Consequently, FibrilJ may erroneously interpret a single fibril as multiple entities, reporting the lengths of various segments rather than the entire fibril. The fibril properties were then exported to an Excel sheet, listing IDs for each fibril detected and measured by FibrilJ. To enhance the accuracy of the length data, we manually reviewed the length data for the fibrils, identifying and correcting falsely detected fibrils and merging different fibril IDs that constitute a single fibril.

To improve the precision of fibril length measurements, we opted for images with lower density and minimal fibril overlap, along with areas of optimal negative (or positive) staining. This approach facilitates more uniform image binarization and minimizes the risk of fibril bisection due to variations in stain intensity across the image. Data from three fibril replicates within each group were aggregated for intergroup comparison. The Kolmogorov–Smirnov test was employed to assess the length and diameter distributions across different fibril types.

## Data Availability

Cryo-EM maps and atomic coordinates have been deposited in the EMDB and PDB with accession codes: EMD-52014 and PDB ID 9HBB. All other data supporting the findings of this study are available from the corresponding author upon reasonable request.

## Supporting Information Available

Detailed experimental protocols and compound characterization (PDF)

## Abbreviations

Ac: acetyl
Acm: acetamidomethyl
AD: Alzheimer’s disease
Bn: benzyl
CBD: corticobasal degeneration
CC: complex coacervation
CD: circular dichroism
cryo-EM: cryo-electron microscopy
CTE: chronic traumatic encephalopathy
DIC: diisopropylcarbodiimide
DIPEA: diisopropylethylamine
DMSO: dimethyl sulfoxide
DPDS: diphenyl diselenide
DSL: diselenide-selenoester ligation
ELISA: enzyme-linked immunosorbent assay
Fmoc: fluorenylmethyloxycarbonyl
FRAP: fluorescence recovery after photobleaching
FTDP-17: frontotemporal dementia with parkinsonism-17
GGT: globular glial tauopathy
GlcNAc: *N*-acetylglucosamine
Gnd: guanidine
HATU: *O*-(7-azabenzotriazol-1-yl)-*N*,*N*,*N*′,*N*′-tetramethyluronium hexafluorophosphate
HEPES: 4-(2-hydroxyethyl)-1-piperazineethanesulfonic acid
HMPB: 4-(4-hydroxymethyl-3-methoxyphenoxy)butyric acid, polymer-bound
HPLC: high-performance liquid chromatography
LC-HRMS: liquid chromatography–high-resolution mass spectrometry
LLPS: liquid–liquid phase separation
Me: methyl
MPAA: mercaptophenylacetic acid
NCL: native chemical ligation
NFTs: neurofibrillary tangles
Oxyma: ethyl cyanoglyoxylate-2-oxime
P(*n*-Bu)_3_: tri-*n*-butylphosphine
PEG: polyethylene glycol
PHFs: paired helical filaments
PMB: *para*-methoxybenzyl
PSP: progressive supranuclear palsy
PTMs: post-translational modifications
RNA: ribonucleic acid
SPPS: solid-phase peptide synthesis
TFA: trifluoroacetic acid
TEM: transmission electron microscopy
Thz: thiazolidine
WT: wild type
